# Preliminary Evaluation of the Safety and Immunogenicity of an Antimalarial Vaccine Candidate Modified Peptide (IMPIPS) Mixture in a Murine Model

**DOI:** 10.1155/2019/3832513

**Published:** 2019-12-30

**Authors:** Jennifer Lambraño, Hernando Curtidor, Catalina Avendaño, Diana Díaz-Arévalo, Leonardo Roa, Magnolia Vanegas, Manuel E. Patarroyo, Manuel A. Patarroyo

**Affiliations:** ^1^Fundación Instituto de Inmunología de Colombia (FIDIC), Bogotá, Colombia; ^2^Master's Programme in Biochemistry, Medical School, Universidad Nacional de Colombia, Bogotá, Colombia; ^3^Faculty of Animal Science, Universidad de Ciencias Aplicadas y Ambientales (U.D.C.A), Bogotá, Colombia; ^4^School of Medicine and Health Sciences, Universidad del Rosario, Bogotá, Colombia; ^5^Pathology Department, Medical School, Universidad Nacional de Colombia, Bogotá, Colombia

## Abstract

Malaria continues being a high-impact disease regarding public health worldwide; the WHO report for malaria in 2018 estimated that ~219 million cases occurred in 2017, mostly caused by the parasite *Plasmodium falciparum*. The disease cost the lives of more than 400,000 people, mainly in Africa. In spite of great efforts aimed at developing better prevention (i.e., a highly effective vaccine), diagnosis, and treatment methods for malaria, no efficient solution to this disease has been advanced to date. The Fundación Instituto de Inmunología de Colombia (FIDIC) has been developing studies aimed at furthering the search for vaccine candidates for controlling *P. falciparum* malaria. However, vaccine development involves safety and immunogenicity studies regarding their formulation in animal models before proceeding to clinical studies. The present work has thus been aimed at evaluating the safety and immunogenicity of a mixture of 23 chemically synthesised, modified peptides (immune protection-inducing protein structure (IMPIPS)) derived from different *P. falciparum* proteins. Single and repeat dose assays were thus used with male and female BALB/c mice which were immunised with the IMPIPS mixture. It was found that single and repeat dose immunisation with the IMPIPS mixture was safe, both locally and systemically. It was observed that the antibodies so stimulated recognised the parasite's native proteins and inhibited merozoite invasion of red blood cells *in vitro* when evaluating the humoral immune response induced by the IMPIPS mixture. Such results suggested that the IMPIPS peptide mixture could be a safe candidate to be tested during the next stage involved in developing an antimalarial vaccine, evaluating local safety, immunogenicity, and protection in a nonhuman primate model.

## 1. Introduction

Malaria represents one of the greatest public health problems worldwide. According to the World Health Organization (WHO), ~219 million new malaria-related cases occurred in 2017 accompanied by ~435,000 deaths. The African continent was the most affected region in the world (92% of cases and 93% of deaths) [1]. The *Global Technical Strategy for Malaria 2016-2030* (WHO) has suggested reducing malarial incidence and mortality by at least 90% and eliminating it in at least 35 countries by 2030 through prevention, diagnosis, and treatment strategies [2].

No significant progress has been observed to date regarding the reduction of cases of malaria worldwide despite the differing strategies used for combating this disease (using insecticide-impregnated mosquito nets for controlling the vector, chemoprophylaxis, and case management) [1, 2]. The most recurrent problem is concerned with the increase in strains which are resistant to antimalarial drugs and insecticide-resistant mosquitoes; this has necessitated the development and combined use of new control and prevention methods, especially a vaccine having high protection capability as time elapses [1, 3].

The Fundación Instituto de Inmunología de Colombia (FIDIC) has thoroughly demonstrated the feasibility of a chemically synthesised, multistage, multiantigen, minimum subunit-based (~20 amino acid-long peptide) vaccine by following a completely functional approach [4, 5]. This has led to ascertaining that peptides derived from the main proteins participating in merozoite (Mrz) invasion of RBCs [6] specifically bind to human RBCs and that sporozoites (Spz) invading hepatic cells [7, 8] bind to the HepG2 hepatocellular carcinoma cell line [9–12].

Immunogenicity and protection assays in *Aotus* monkeys have shown that high activity binding peptides (HABPs) [12] having a conserved sequence (cHABP) have not induced an immune response, suggesting that despite the importance of their biological role, they are immunologically silent [4, 5]. By contrast, HABPs having a variable sequence (vHABPs) have induced a nonprotective immune response (or only a short-term one) [4, 13], an immune evasion mechanism for these sequences (smokescreens distracting the immune response) [14–16]. However, when some cHABP residues [17, 18] have been replaced by amino acids (aa) having similar mass and volume, but different polarity, modified analogues (mHABPs) have been seen to induce a protective immune response in *Aotus* monkeys against experimental challenge [5, 13, 19–22].

Nuclear magnetic resonance (NMR) and *in silico* structural binding studies have shown that mHABPs having polyproline II (PPIIL) helix structures [23, 24] can bind to HLA-DR*β*1∗ molecules covering most MHC-II allele variants [21, 25, 26] and have greater interaction with the T cell receptor (TCR). This would suggest the stable formation of the MHC-II-mHABP-TCR trimer complex and thus the capability for inducing a protective immune response [27–31]. Protection-inducing mHABPs have thus been called *Pf* immune protection-inducing protein structures (IMPIPS) in view of their close structure-protection relationship [32, 33].

This study thus used a murine model for evaluating the immunogenicity, local toxicity, and systemic toxicity [34–38] of a mixture of 23 IMPIPS. These were derived from the main *P. falciparum* Spz (circumsporozoite protein 1 (CSP-1), thrombospondin-related anonymous protein (TRAP), sporozoite threonine and asparagine-rich protein (STARP), sporozoite microneme proteins essential for cell traversal (SPECT-1 and SPECT-2), cell-traversal protein for ookinetes and sporozoites (CelTOS), and sporozoite invasion-associated protein 1 and 2 (SIAP-1 and SIAP-2)) [9, 11, 39, 40], as well as Mrz proteins (apical membrane antigen-1 (AMA-1), erythrocyte-binding protein 175 (EBA-175), erythrocyte-binding protein 140 (EBA-140), serine repeat antigen (SERA-5), merozoite surface protein-1 (MSP-1), and histidine-rich protein II (HRP-II)) [10, 39, 40]. Previous studies testing these peptides individually have shown that the antibodies induced were able to recognise the original template protein when expressed as a recombinant (Supplementary [Supplementary-material supplementary-material-1]).

## 2. Materials and Methods

### 2.1. Peptide Synthesis and Purification

Twenty-three polymer peptides (Table 1) were modified following previously reported principles [32, 33, 40, 41] to render them immunogenic and then synthesised using solid-phase multiple peptide synthesis following the tert-butyloxycarbonyl (t-Boc) synthesis strategy described by Merrifield [42] and modified by Houghten [43]. All peptides were derived from fully conserved and functionally relevant regions of the corresponding proteins. Such a Merck-Hitachi L-6200 A chromatograph (Merck) fitted with a UV-VIS L-4250 210 nm wavelength detector was used for determining synthesised peptide purity by high-performance reversed-phase liquid chromatography. A Microflex mass spectrometer (Bruker Daltonics) was then used for characterising them by matrix-assisted laser desorption/ionisation-time of flight (MALDI-TOF).

### 2.2. Animals

Forty BALB/c mice (20 males and 20 females) were used for evaluating the formulation's safety and immunogenicity. The mice were aged 5 to 6 weeks when they were immunised, according to WHO recommendations [37]. The mice were acquired from the Universidad Nacional de Colombia's Faculty of Animal Science's Biotherium.

#### 2.2.1. Single Dose Local Tolerance

This assay enabled determining possible inflammatory reactions at the different treatments' inoculation sites [37]; this involved using 18 BALB/c mice (9 males and 9 females), following the protocols established by the regulatory authorities [34–37]. The animals were randomly assigned to 3 groups (Table 2), each consisting of 3 males and 3 females, in line with the principles of reduction and refinement [44].

The animals were immunised by subcutaneous (SC) route at the base of the tail with 100 *μ*L of the formulation. The animals were observed twice a day after they had been immunised for detecting changes in their behaviour, signs of disease, or toxicity. The injection site and the tissue around it were examined 1, 3, 24, 48, and 72 hours after immunisation to ascertain the presence of erythema, oedema, eschar, and necrosis; the parameters described by Cox were used for evaluating their degree [45]. The animals were anesthetised with ketamine (80-120 mg/kg) and xylazine (5-16 mg/kg) by intraperitoneal (IP) route on the third day and sacrificed by cervical dislocation.

#### 2.2.2. Repeat Doses

Twenty-two BALB/c 5- to 6-week-old mice (11 males and 11 females) were randomly distributed into three groups for evaluating possible toxic reactions produced by repeat inoculations (SC) of the *Pf*-IMPIPS peptide mixture (30 *μ*g in total). Toxicity due to repeat doses can occur as a result of repeat administration of a product over a specific period [35–37] (Table 3).

**Figure 1:**
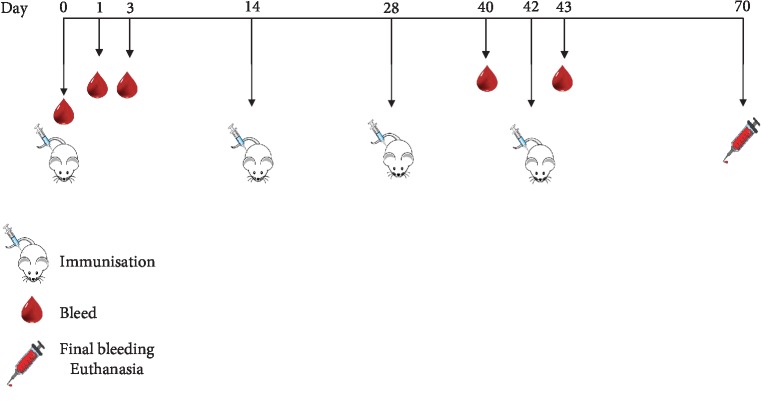
Immunisation scheme for evaluating local tolerance and systemic toxicity due to repeat doses in mice.

The animals were immunised 4 times with a 14-day interval as the amount of doses in an animal model must be equal to or greater than the amount of doses for clinical assays [37], and it would be expected that the amount of doses administered would not exceed two in clinical assays (Figure 1).

Each group was immunised with 100 *μ*L of the formulation on days 0, 14, 28, and 42; the formulation was administered by SC route at the base the tail. The immunisation sites were examined 1, 3, and 24 h after each injection looking for signs of erythema, oedema, eschar, and necrosis.

The animals were observed twice per day for evidence of any adverse reaction to the injection or the presence of disease, and a weekly physical examination was made for monitoring every animal's overall state of health. Their weight and food consumption were also monitored before beginning the immunisation protocol and after immunisation on days 0, 3, and 7 and every week thereafter until day 70.

Mouse body temperature was measured with an infrared thermometer (Benetech GM320) before and after each immunisation (0, 4, and 24 h) at five different sites on their abdomens. The average of five readings was recorded [46].

Blood samples were taken from the facial vein before immunisation and on days 1, 3, 40, 43, and 70 following the first immunisation to rule out acute and chronic alterations and in case of any abnormal findings. These samples were used for evaluating blood urea nitrogen (BUN), creatinine (CRE), haematocrit (HCT), red blood cell (RBC) count, white blood cell (WBC) count, and total plasma protein (TPP) levels. The animals were anesthetised with ketamine (80-120 mg/kg) and xylazine (5-16 mg/kg) on day 70 by IP route and sacrificed by cervical dislocation [47].

The mice were necropsied, and kidney, heart, duodenum, spleen, and liver samples were taken for histological study to evaluate possible damage. The samples were kept in 10% formaldehyde and processed and analysed at the Universidad de Ciencias Aplicadas y Ambientales (U.D.C.A) pathology laboratory.

### 2.3. Immunogenicity

#### 2.3.1. Indirect Immunofluorescence Assay (IFA)

The indirect immunofluorescence assay (IFA) was used for evaluating an antibody's ability to recognise the parasite's native proteins, according to a previously reported methodology [48]. Briefly, RBC infected with mature schizont-stage parasites were taken from a *P. falciparum* FCB2 strain continuous culture synchronised with 5% sorbitol, at 5% to 7% parasitaemia. The RBCs were washed three times with phosphate-buffered saline (PBS) (7.2 to 7.4 pH) and spun at 1,200 g for 5 min. The pellet was suspended in filtered PBS until reaching 1% final dilution, 20 *μ*L/well of this suspension was seeded on 8-well slides and left to settle for 20 minutes, and the supernatant was collected. The slides were left to dry at room temperature (RT); they were then blocked with 30 *μ*L/well PBS-1% skimmed milk for 10 min at (RT), washed once with PBS (7.2 to 7.4 pH) for 5 min, and left to dry.

Then, in duplicate, 2.5 *μ*L/well serum from the final bleeding was seeded on 8-well slides at 1 : 20 dilution in PBS. The slides were incubated in a moist chamber for 30 min, washed with PBS (7.2 to 7.4 pH) six times for 5 min each wash, and left to dry. This was followed by placing 10 *μ*L/well fluorescein isothiocyanate- (FITC-) labelled anti-mouse IgG (Vector Laboratories, Inc.) in each well at 1 : 20 dilution in PBS as well as 1 : 80 4 *μ*L/well Evans blue to reduce background and increase contrast in the imaging/reading. The slides were incubated in a moist chamber in the dark for 30 min, washed, and left to dry. The slides were observed with a fluorescence microscope (Olympus B51) at 1,000x magnification. The assay was made by pooling all the sera from each group due to the low serum volume available. Serum from a *P. berghei*-infected mouse was used as the positive control. The immunofluorescence signal was analysed semiquantitatively in the photographs already taken as follows: (+++) corresponds to the maximum fluorescence (positive control), (0+) to the negative control, (++) to IMPIPS plus adjuvant, and (+) to IMPIPS plus PSS.

#### 2.3.2. Invasion Inhibition Assay

The *P. falciparum* FCB-2 strain (parasite ring stage) culture, previously synchronised with 5% sorbitol, 1-8 h postinvasion, was used for determining the ability of serum from the mice immunised with the IMPIPS mixture to inhibit Mrz invasion of erythrocytes [49]. A 384-well plate [50] seeded with 1.7 *μ*L/well parasite culture (2% haematocrit and 0.1% parasitaemia) was incubated with 10 *μ*L/well of serum from the final bleeding which had been inactivated (preimmune and postimmune) at 20%, 10%, 5%, and 2.5% concentrations (%*v*/*v*). Each well's final volume was 50 *μ*L, which was completed with RPMI 1640 media (Gibco); each sample was analysed in duplicate. The plate was incubated at 37°C for 48 h in a 5% O_2_, 5% CO_2_, and 90% N_2_ atmosphere. Parasitised RBCs (pRBCs) and sera from control group mice (day 0) were used as the negative control, whilst human RBCs with chloroquine (150 nM) were used as the positive control for the invasion inhibition assay. Parasite culture supplemented with healthy human plasma was used as the culture control. The plate was spun at 1,800 rpm after 48 h incubation, and the culture supernatants were removed; the cells were then labelled with 1X 50 *μ*L SYBR Green (1 : 10,000) (Invitrogen) for 30 min in the dark. Cell suspensions were washed three times with PBS and analysed by flow cytometry (FACSCanto II, Becton Dickinson). FlowJo 7.5 (Tree Star, Inc.) was used for analysing the data [51]. The assay was made by pooling all the sera from each group due to the low serum volume available.

### 2.4. Statistical Analysis

GraphPad Prism 7 software was used for statistically analysing the data for each group of animals (minimum and maximum values, the mean and standard deviation (SD)). The Shapiro-Wilk test of normality was used for comparing the groups of animals according to treatment; ANOVA was then used for analysing normally distributed data, whilst nonnormally distributed data was analysed by the Tukey or Kruskal-Wallis multiple comparison test and Dunn's multiple comparison test. Differences were considered statistically significant at *p* < 0.05. Stata software's linear regression model was used for statistically analysing histopathological results, using Pearson's *Χ*^2^ test, for determining whether the IMPIPS mixture was toxic for the organs and tissues analysed here.

### 2.5. Ethical Statements

The mice were maintained according to the bioethical regulations laid down in Colombian Law 84/1989 [52], Colombian Ministry of Health resolution 8430/1993 [53], and the *Guide for the Care and Use of Laboratory Animals* of the National Institutes of Health [54]. Regulations stipulated by the American Veterinary Medical Association's (AVMA) Panel on Euthanasia (2013) were also considered [47]. All the animals were fed on Rodent Diet 5010 (LabDiet) and provided with water *ad libitum*. The Universidad de Ciencias Aplicadas y Ambientales (U.D.C.A) ethics committee, regulated by Agreement 285/2008, Chapter VII, endorsed this research.

## 3. Results

### 3.1. Local Tolerance: Single Dose in Mice

No deaths occurred in any of the groups being studied for this assay. None of the animals (females or males) in the different groups treated and evaluated 1, 3, 24, 48, and 72 h postimmunisation had lesions at the inoculation site, compared to the control group (not immunised).

### 3.2. Local and Systemic Tolerance: Repeat Doses in Mice

There were no deaths in any of the groups of animals when evaluating local toxicity due to repeat doses of the IMPIPS mixture. No local adverse reactions were observed (postimmunisation), such as erythema, oedema, eschar, or necrosis, at the inoculation site in any of the groups.

#### 3.2.1. Physiological Parameters

The Shapiro-Wilk test of normality confirmed a normal distribution for male and female weight values. Average weight gain according to the means for each experimental group and the SD (mean ± SD) obtained for each treatment for male mice immunised with PSS, IMPIPS+PSS, and MPIPS+adjuvant was 25.32 ± 2.56 g, 25.04 ± 3.06 g, and 23.34 ± 3.38 g, respectively, whilst for the females immunised with just PSS, IMPIPS+PSS, and IMPIPS+adjuvant, this was 19.70 ± 1.99 g, 19.23 ± 2.39 g, and 19.38 ± 3.01, respectively (Supplementary [Supplementary-material supplementary-material-1]). Statistical analysis revealed no significant differences between the groups (*p* > 0.05).

Mean weekly consumption of food by the males immunised with PSS, IMPIPS+PSS, and MPIPS+adjuvant was 30.69 ± 1.43 g, 30.2 ± 1.25 g, and 30.97 ± 2.99 g, respectively, compared to the females immunised with PSS, IMPIPS+PSS, and MPIPS+adjuvant (24.05 ± 1.25 g, 24.88 ± 1.20 g, and 25.27 ± 1.72 g, respectively) (Supplementary [Supplementary-material supplementary-material-1]). Statistical analysis did not reveal any significant differences between the groups (*p* > 0.05).

The temperature of male mice immunised with PSS, IMPIPS+adjuvant, and IMPIPS+PSS ranged from 30.72°C to 32.27°C, 29.56°C to 32.63°C, and 30.57°C to 32.23°C, respectively, during the study, whilst for females immunised with PSS, IMPIPS+adjuvant, and IMPIPS+PSS, it ranged from 31.05°C to 37.57°C, 29.62°C to 33.91°C, and 31.2°C to 33.35°C, respectively (Supplementary [Supplementary-material supplementary-material-1]). Overall, temperatures remained between the minimum and maximum ranges of the control group.

#### 3.2.2. Haematological Parameters

The haematic picture evaluated parameters related to erythrocytes (erythrocyte count (RBC), haematocrit (HCT), haemoglobin (Hb), and total plasma proteins (TPP)) and leukocytes (leukocyte (WBC) count) for determining possible alterations caused by the formulation. The reference values for analysing each biochemical parameter were determined by mean control ± SD; no significant differences (*p* > 0.05) were observed regarding either erythrocytes or leukocytes. Complete blood cell count (CBC) values came within the stated parameters, except for day 3 when control group females had a slight reduction in haematocrit and haemoglobin, possibly due to previous bleedings. By contrast, an increase in leukocytes was observed in control group males; this increase could have been caused by stress due to the bleeding.

**Figure 2:**
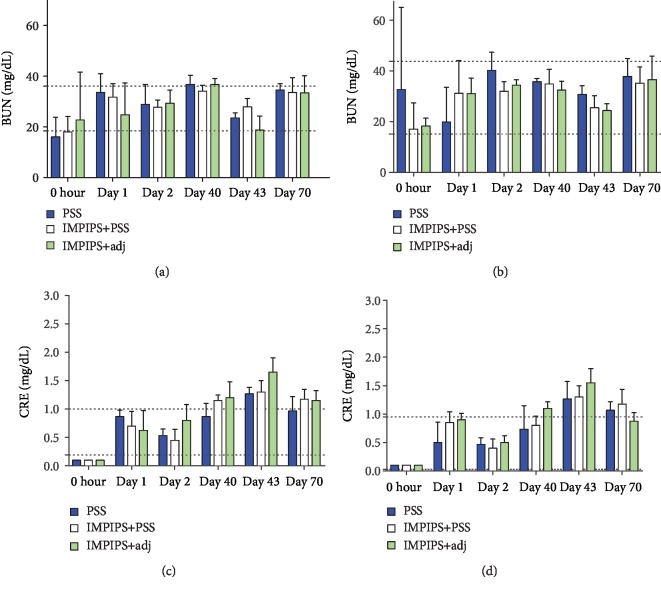
Means for the biochemical parameters: blood urea nitrogen (BUN) and creatinine (CRE). BUN and CRE values are shown for females (a and c) and males (b and d), according to time elapsed and group immunised. The horizontal dotted lines indicate the reference values.

Renal function was evaluated by measuring BUN and CRE. ANOVA analysis identified no statistically significant differences between the different treatments when comparing values between the groups or when comparing control group values (saline solution) to those for the other study groups (*p* > 0.05) (Figure 2). The reference values for analysing each biochemical parameter were determined from the means for the controls ± SD (Figure 2).

#### 3.2.3. Histopathology

**Figure 3:**
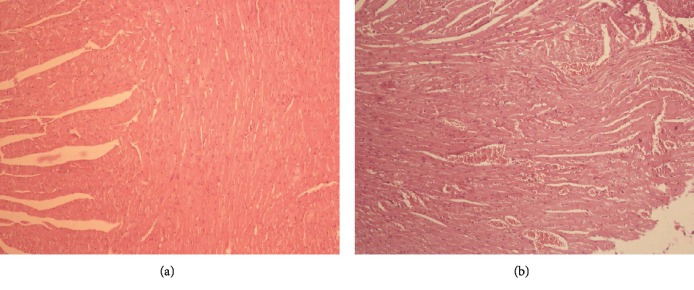
Histological section of the myocardium on day 70, stained with haematoxylin-eosin: (a) histology for the normal myocardium in a mouse treated with physiological saline solution (100x); (b) section of the myocardium from a mouse belonging to the group immunised with IMPIPS+adjuvant; microcirculatory changes related to moderate vascular congestion were observed (100x).

Microcirculatory changes related to slight and moderate vascular congestion were observed in the myocardium in 2/4 females immunised with the IMPIPS+adjuvant formulation; there was no evidence of congestion in the other animals from the same group or from the other groups (*p* > 0.05). No microcirculatory changes were seen in any of the immunised animals, such as oedema and/or haemorrhage, inflammatory infiltrate, structural changes, or binucleation (Figure 3).

**Figure 4:**
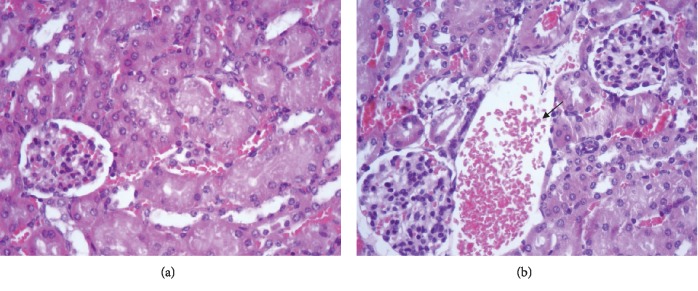
Histological section of the kidney on day 70, stained with haematoxylin-eosin: (a) histology for the normal kidney in a mouse treated with physiological saline solution (400x); (b) section of the kidney from a mouse from the group immunised with IMPIPS+adjuvant had microcirculatory changes regarding slight congestion (arrow) (400x).

Slight congestion was observed in the kidneys of at least one animal from every group. These changes occurred more in females than in males, since a lesion was found in just one male compared to 6 females (2 from each group) in which congestion was observed. No microcirculatory changes such as oedema and haemorrhage, inflammatory infiltrate, structural changes, or binucleations were observed in any of the study groups (Figure 4).

**Figure 5:**
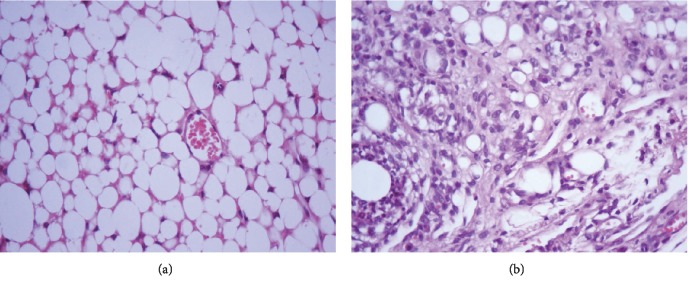
Histological section of the mouse mesentery on day 70, stained with haematoxylin-eosin: (a) histology for the normal mesentery of a mouse treated with saline solution (400x); (b) mesentery having mixed inflammatory infiltrate from a mouse treated with IMPIPS+adjuvant (400x).

**Figure 6:**
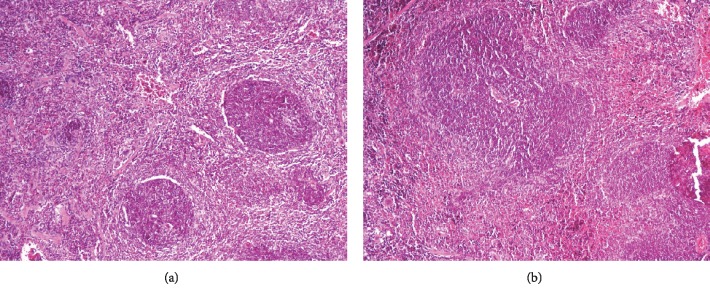
Histological section of mouse lymphoid tissue on day 70, stained with haematoxylin-eosin: (a) histology of mouse normal lymphoid tissue treated with saline solution (100x); (b) lymphoid tissue from a mouse treated with IMPIPS+adjuvant having nodular hyperplasia (100x).

No macroscopic or microscopic alterations were observed when analysing the duodenum, though 50% of the mice immunised with IMPIPS+adjuvant had mixed inflammatory infiltrate in the mesentery (Figure 5). Likewise, follicular hyperplasia was observed in 100% of the mice immunised with IMPIPS+adjuvant when analysing lymphoid tissue, 50% being slight and 50% moderate. 37.5% of the mice immunised with IMPIPS+PSS had this reaction to a slight degree and 25% to a moderate degree. Two (33.3%) control group mice had slight follicular hyperplasia (Figure 6). A scale was generated to semiquantify follicular hyperplasia according to the number of nodules using a magnification of 400x (Supplementary [Supplementary-material supplementary-material-1]).

### 3.3. Immunogenicity

#### 3.3.1. Determining Anti-IMPIPS Serum Ability to Recognise *P. falciparum*-Infected RBC by IFA

**Figure 7:**
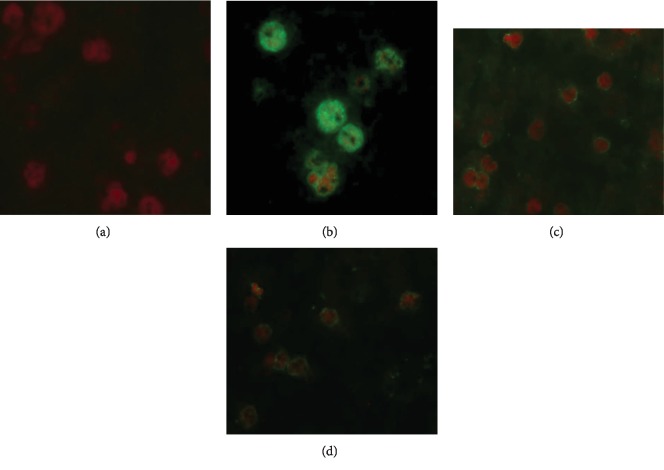
Immunofluorescence assay with mouse anti-IMPIPS antibodies in *Plasmodium falciparum*-infected erythrocytes (FCB2 strain). Results were analysed semiquantitatively according to fluorescence intensity from null (0+) to maximum fluorescence (+++). (a) PBS (negative control) (0+). (b) Serum from a *P. berghei*-infected mouse (positive control) (+++). (c) Serum from the group of mice immunised with IMPIPS plus adjuvant (++). (d) Serum from the group immunised with IMPIPS plus PSS (+). Each study was done in duplicate. Mouse serum and the FITC-labelled anti-mouse IgG (green fluorescence) were used at 1 : 20 dilution. The samples were analysed with a fluorescence microscope (Olympus B51) with an immersion objective (1,000x).

An indirect immunofluorescence assay was used for determining anti-IMPIPS serum ability to recognise pRBC. The serum from mice immunised with IMPIPS+adjuvant as well as that from those immunised with IMPIPS+PSS was able to recognise pRBC (Figure 7). Considering that some *P. berghei* proteins share high identity with their *P. falciparum* counterparts, such as enolase [55], the higher fluorescence intensity observed in the positive control might be due to the higher number of proteins being recognised versus just the six blood-stage proteins being recognised from animals immunised with the peptide mixture.

#### 3.3.2. Determining Anti-IMPIPS Antibodies' Merozoite Invasion Inhibition Capability

**Figure 8:**
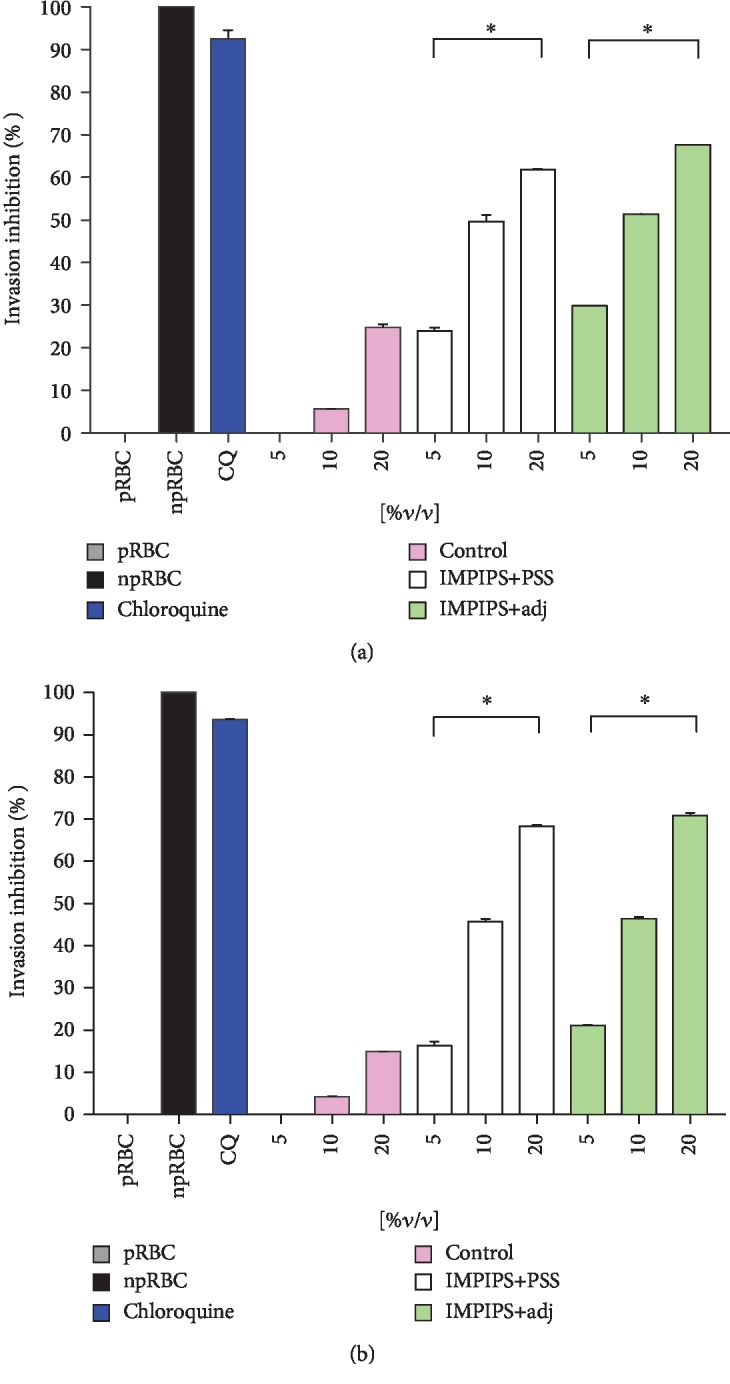
Percentage invasion inhibition of anti-IMPIPS serum. (a) Males. (b) Females. The assay was done in duplicate in three different experiments. Different serum concentrations were used (5, 10, and 20%*v*/*v*); pRBC (C-), npRBC (C+), and chloroquine (C+) were used as controls of the test. Control group mice (day 0) were used as the positive control of invasion (pink). Significance was determined at ^∗^*p* < 0.05.

The functional role of antibodies stimulated by immunisation with IMPIPS was determined by an *in vitro* invasion inhibition assay. The serum from animals immunised with the IMPIPS peptide mixture+PSS was able to inhibit invasion, maximum values being 61.84% for males and 68.34% for females. Likewise, the serum from the animals immunised with the IMPIPS peptide mixture+adjuvant had 67.62% maximum invasion inhibition values for males and 70.82% for females. It was found that inhibition was concentration dependent (*p* < 0.05). No statistical difference was observed between inhibition percentages for the females compared to those for the males (*p* > 0.05) (Figure 8). Sera surpassing 70% were considered strong inhibitors, whilst those ranging from 50% to 69% were considered medium-high inhibitors. Those having 30% to 49% were considered medium-low inhibitors, those from 10%-29% are low inhibitors, and those < 9% were considered negative.

## 4. Discussion

Toxicological studies of the formulation to be used in clinical studies are of the utmost importance when developing vaccines as they provide information about possible adverse effects which might arise due to the formulation, either at the inoculation site or in the different organs and tissues of subjects being vaccinated [37, 38]. This study thus evaluated the safety and immunogenicity of a mixture of 23 modified peptides derived from 8 Spz proteins (CSP-1, TRAP, STARP, SPECT-1 and SPECT-2, CelTOS, and SIAP-1 and SIAP-2) and 6 Mrz proteins (AMA-1, EBA-175, EBA-140, SERA-5, MSP-1, and HRP-II) [9–11] in a murine model as a synthetic antimalarial vaccine candidate.

The single dose local tolerance study was aimed at evaluating the site exposed to the formulation 72 h postimmunisation; no adverse reactions such as erythema, oedema, eschar, or necrosis were observed in the mice immunised with the IMPIPS mixture+PSS or in those immunised with IMPIPS+adjuvant. This suggested that the IMPIPS mixture did not produce local toxic effects due to single dose SC immunisation. The forgoing led to continuing local and systemic tolerance studies regarding repeat doses (4 immunisations) where SC immunisation also did not produce adverse effects such as erythema, oedema, eschar, or necrosis at the administration site, suggesting that repeat IMPIPS doses did not produce irritation or toxicity at the immunisation site.

Male and female mice immunised with the IMPIPS peptide mixture+PSS or IMPIPS+adjuvant gained weight and increased their weekly food consumption, and their body temperature was within established parameters (i.e., regarding the formulation's systemic effects) [56], thereby supporting the idea that the IMPIPS mixture did not affect physiology.

Blood chemistry analysis showed that creatinine values did not exceed the parameters compared to control values during the first bleeding. Creatinine values on day 40 exceeded the parameters; however, they became reduced by day 70, coming within normal parameters for the males. Such transitory increase could have been due to stress or dehydration since, unlike other mammals, mice excrete creatinine in their urine. Once the situation had become resolved, creatinine returned to its normal values [56]. BUN values came within normal parameters. Since no other damage was observed on day 70, the histological study of the kidneys verified that an increase in creatinine was due to prerenal causes and not to renal damage.

Regarding histological analysis, the microcirculatory changes in the myocardium compatible with congestion did not arise from administering the IMPIPS peptide mixture, since control group animals also had this pathology. Such changes could mainly have been due to the hypovolemic shock caused by the final bleeding; this would have occurred because haemorrhagic shock affects tissue perfusion [57].

Mixed inflammatory infiltrate was observed in the histological study of the mesentery; this description refers to localised accumulations of mononuclear and polymorphonuclear cells, indicating the presence of a foreign body in acute phase, i.e., causing the antigenic stimulus to continue. This finding in the animals immunised with IMPIPS+adjuvant and not in those immunised with IMPIPS+PPS or in the control group indicated that the antigen continued being active. This could have been caused by the formation of a deposit at the injection site due to the adjuvant's mechanism of action (doses 2, 3, and 4 of the formulation were administered by IP route) [58].

The nodular hyperplasia observed in animals' lymphoid tissue is mainly due to normal lymphoid nodule inflammation in response to an antigen. Such response in this case was triggered by the immunisation; such reaction is also known as reactive lymphoid hyperplasia [59] which, as expected, was much stronger in the animals immunised with the formulation containing IMPIPS+adjuvant.

FIDIC's previous studies have shown that individual immunisation of IMPIPS in *Aotus* monkeys has stimulated the production of antibodies which have been able to recognise parasite proteins in their native form and induce a protective immune response, determined by the total absence of parasites in the blood following experimental challenge [30, 32, 33]. The present study highlighted the fact that serum from male and female mice immunised with the IMPIPS mixture+adjuvant or IMPIPS+PSS recognised the parasite in the Mrz stage. This indicated that although the peptides had been modified for their presentation by human MHC-II [27, 29, 31], the mixture was capable of inducing an immune response against the native proteins from which they were derived, even in a murine model, thereby reinforcing the idea of using IMPIPS in an antimalarial vaccine [60]. Such response was seen in the immunofluorescence and invasion inhibition assays.

## 5. Conclusions

Local tolerance and systemic safety tests regarding single and repeat doses in this study showed no toxicity induced by the IMPIPS mixture in a murine model 70 days after the first immunisation, reaffirming that peptide-based vaccines can represent a safe option. The IMPIPS mixture was immunogenic in a murine model, even when the peptides were designed for human MHC-II. Such results suggested that the IMPIPS mixture is safe and thus further immunogenicity and protection assays in a nonhuman primate model such as the *Aotus* spp. monkey but delivered with adjuvants authorised for human use are recommended.

## Figures and Tables

**Table 1:** List of peptides (IMPIPS) included in the mixture.

mHABP	Sequence	Protein	Theoretical mass (kDa)	Mass (*m*/*z*)
32958	CGGNGNGQGLNMNNPPNFNVDENAGC	CSP	2,436.8	2,436.9
25608	CGKNSFSLGENPNANPGC	CSP	1,809.3	1,807.1
24312	CGDLGHVNGRDTMNNIVDENKYGC	TRAP	2,715.4	2,713.3
24242	CGVWDEWSPVSTAVGMGTRSRKGC	TRAP	2,568.8	2,567.3
24250	CGKSLDIERKMADPQAQDNNGC	TRAP	2,393.8	2,392.2
24254	CGGAATPYSGEPSPFDEVLGEEGC	TRAP	2,372.9	2,373.2
24320	CGVIKHMRFHADYQAPFLGGGYGC	STARP	2,628.8	2,626.0
38150	CGTDLILKALGKLQNTNKGC	SPECT	2,090.9	2,089.3
38890	CGSDYTKALAAEAKVSYWGIGC	SPECT-2	2,435.1	2,436.2
38128	CGKLTPISDSFDSDDTKESYDKGC	SPECT-2	2,612.3	2,611.7
38976	CGVDTTIWSGVNNLSHVALDGGC	SPECT-2	2,316.0	2,316.3
38880	CGETAVGALQADEIWNYNTGC	CELTOS	2,212.9	2,213.9
38162	CGKTQGHSYHLRRKNGVKHPVYGC	SIAP-1	2,726.6	2,729.2
38884	CGGLHYSTDSQPNLDISFGELGC	SIAP-2	2,411.0	2,411.3
13486	CGMIKASFDPTGAFKSPRYKSHGC	AMA-1	2,589.6	2,588.8
37206	CGNDKLYFDEYWKVIKKDGC	EBA-175	2,425.2	2,405.3
24292	CGLTNQNINIDQEFNLMKHGFHGC	EBA-175	2,734.4	2,732.0
22690	CGNNIPSRYNLYDKMLDLDGC	EBA-175	2,405.2	2,402.2
36620	CGLKNKETTKDYDMFQKIDSFLGC	EBA-140	2,785.6	2,781.7
22796	CGDNILVKMFKVIENNDKSELIGC	SERA	2,683.6	2,681.7
23426	CGKKVQNLTGDDTADLATNIVGGC	SERA	2,394.0	2,395.4
10014	CGEVLYHVPLAGVYRSLKKQLEGC	MSP-1	2,663.5	2,662.9
24230	CGSAFDDNLTAANAMGLILNKRGC	HRP-2	2,456.4	2,453.4

*m*/*z*: mass-to-charge ratio.

**Table 2:** Single dose: distribution of the groups of mice according to treatment.

	Treatment	Size
Group 1	Physiological saline solution (PSS) (control)	3M+3F
Group 2	IMPIPS mixture (30 *μ*g in total)+PSS (1 : 1)	3M+3F
Group 3	IMPIPS mixture (30 *μ*g in total)+Freund's adjuvant^∗^ (1 : 1)	3M+3F

^∗^The immunisation was made with complete Freund's adjuvant.

**Table 3:** Repeat doses: distribution of the groups of mice according to treatment.

	Treatment	Size
Group 1	Physiological saline solution (PSS) (control)	3M+3F
Group 2	IMPIPS mixture+PSS (1 : 1)	4M+4F
Group 3	IMPIPS mixture+Freund's adjuvant^∗^ (1 : 1)	4M+4F

^∗^The first immunisation was made with complete Freund's adjuvant and those thereafter with Freund's incomplete adjuvant.

## Data Availability

The data used to support the findings of this study are included within the article.

## Supplementary Materials

Supplementary Materials Supplementary Figure 1: physiological parameters for male and female mice: weight, weekly consumption of food, and body temperature values. Dotted lines show upper and lower normal values. Supplementary Table 1: publications referencing peptides included in the present study, in which the antibodies raised recognise the corresponding protein expressed as a recombinant. Supplementary Table 2: semiquantitative scale of follicular hyperplasia using a 400x magnification.
