# Supplementary material for: Preliminary Evaluation of the Safety and Immunogenicity of an Antimalarial Vaccine Candidate Modified Peptide (IMPIPS) Mixture in a Murine Model
Source: J Immunol Res. 2019 Dec 30;2019:3832513. doi: 10.1155/2019/3832513 (PMC7012257; doi:10.1155/2019/3832513)
Supplement: Supplementary Materials — Supplementary Figure 1: physiological parameters for male and female mice: weight, weekly consumption of food, and body temperature values. Dotted lines show upper and lower normal values. Supplementary Table 1: publications referencing peptides included in the present study, in which the antibodies raised recognise the corresponding protein expressed as a recombinant. Supplementary Table 2: semiquantitative scale of follicular hyperplasia using a 400x magnification. [file 3832513.f1.docx]

**Supplementary Figure 1.** Physiological parameters for male and female mice: weight, weekly consumption of food and body temperature values. Dotted lines show upper and lower normal values.


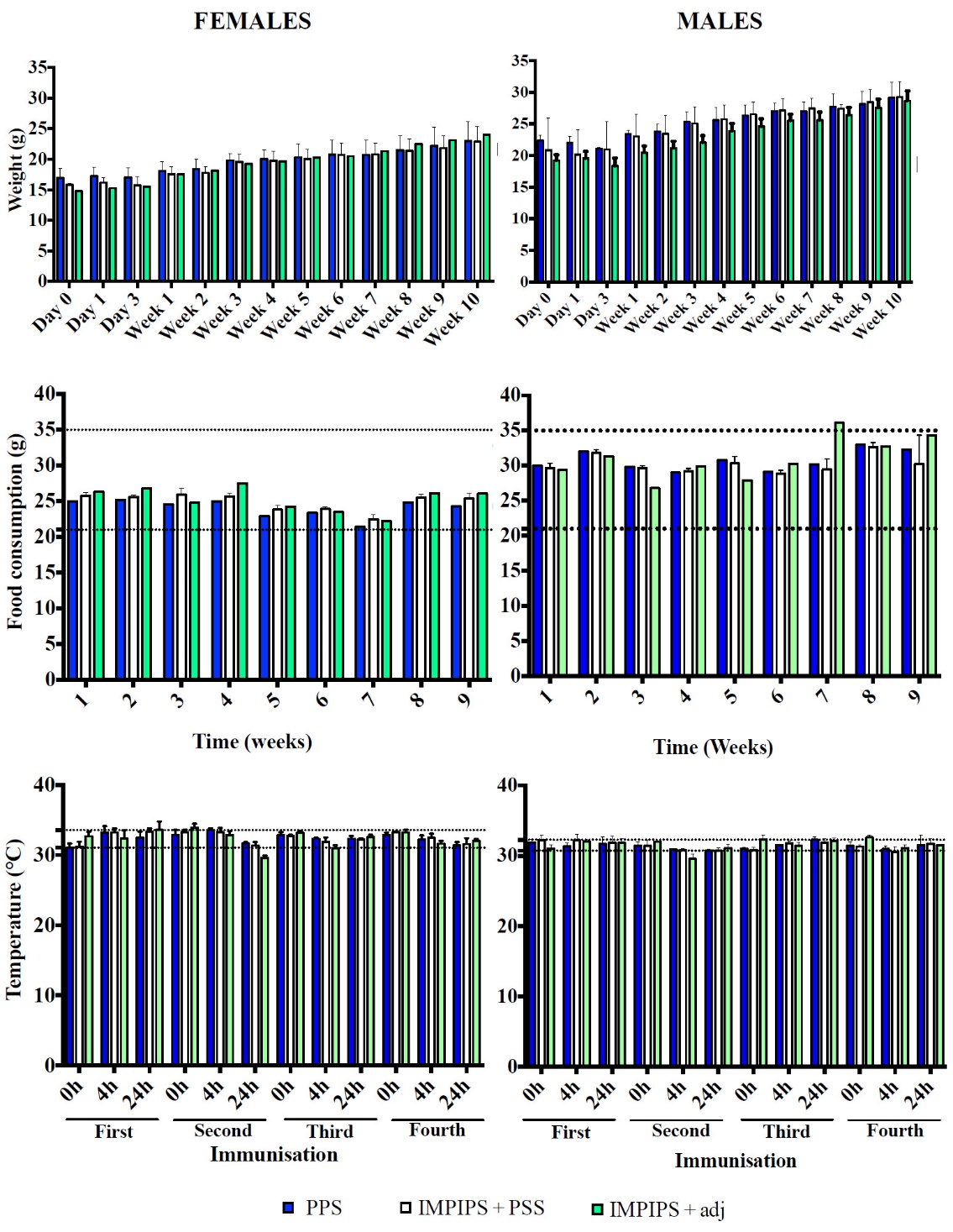


**Supplementary Table 1.** Publications referencing peptides included in the present study, in which the antibodies raised recognise the corresponding protein expressed as recombinant.

| **mHABP** | **Protein** | **Reference** |
| --- | --- | --- |
| 32958 | CSP | [1] |
| 25608 | CSP | [1] |
| 24312 | TRAP | [2] |
| 24242 | TRAP | [3] |
| 24254 | TRAP | [4,5] |
| 24320 | STARP | [2] |
| 24292 | EBA-175 | [5] |
| 23426 | SERA | [6] |
| 10014 | MSP-1 | [7] |
| 24230 | HRP-2 | [7] |

**References**

[1] Patarroyo ME, Bermudez A, Alba MP. The high immunogenicity induced by modified sporozoites’ malarial peptides depends on their phi (φ) and psi (ψ) angles. Biochem Biophys Res Commun 2012;429:81–6.

[2] Curtidor H, Vanegas M, Alba MP, Patarroyo ME. Functional, immunological and three-dimensional analysis of chemically synthesised sporozoite peptides as components of a fully-effective antimalarial vaccine. Curr Med Chem 2011;18:4470–502.

[3] Patarroyo ME, Alba MP, Reyes C, Rojas-Luna R, Patarroyo MA. Malaria parasite’s Achilles’ heel: functionally-relevant invation structures. Curr Issues Mol Biol 2016;18:11–20.

[4] Bermúdez A, Calderon D, Moreno-Vranich A, Almonacid H, Patarroyo MA, Poloche A, et al. Gauche+ side-chain orientation as a key factor in the search for an immunogenic peptide mixture leading to a complete fully protective vaccine. Vaccine 2014;32:2117–26.

[5] Patarroyo ME, Bermúdez A, Alba MP, Vanegas M, Moreno-Vranich A, Poloche LA, et al. IMPIPS: The Immune Protection-Inducing Protein Structure concept in the search for steric-electron and topochemical principles for complete fully-protective chemically synthesised vaccine development. PLoS One 2015;10:1–32.

[6] Patarroyo ME, Moreno-Vranich A, Bermúdez A. Phi (Φ) and psi (Ψ) angles involved in malarial peptide bonds determine sterile protective immunity. Biochem Biophys Res Commun 2012;429:75–80.

[7] Patarroyo ME, Bermudez A, Patarroyo MA. Structural and immunological principles leading to chemically synthesized, multiantigenic, multistage, minimal subunit-based vaccine development. Chem Rev 2011;111:3459–507.

**Supplementary Table 2.** Semi-quantitative scale of follicular hyperplasia using a 400X magnification.

| **Follicular hyperplasia** | **Characteristics** | **NUMBER OF INDIVIDUALS** | | | **TOTAL** |
| --- | --- | --- | --- | --- | --- |
|  |  | **Control** | **IMPIPS+AdJ** | **IMPIPS+PSS** |  |
| Normal | Follicular structures evenly distributed in the cortical region (3-5 lymphoid follicles per field at 400X magnification), without architectural expansion to other zones. | Females: 3 Males: 1 | Females: 0 Males: 0 | Females: 1 Males: 2 | Females: 4 Males: 3 |
| Grade I | Increased number of follicular structures (5-7 lymphoid follicles per field at 400X magnification), with mild increase in lymphocyte number and expansion up to the paracortical border. | Females: 0 Males: 2 | Females: 2 Males: 1 | Females: 2 Males: 1 | Females: 4 Males: 4 |
| Grade II | Increased number of follicular structures (7-8 lymphoid follicles per field at 400X magnification), with moderate increase in lymphocyte number and moderate expansion up to the paracortical and internodular regions. | Females: 0 Males: 0 | Females: 2 Males: 3 | Females: 1 Males: 1 | Females: 3 Males: 4 |
| Grade III | Increased number of follicular structures with severe increase in lymphocyte number; diffuse and severe expansion up to the paracortical and internodular regions. | Females: 0 Males: 0 | Females: 0 Males: 0 | Females: 0 Males: 0 | Females: 0 Males: 0 |
| **TOTAL** |  | **Females: 3 Males: 3** | **Females: 4 Males: 4** | **Females: 4 Males: 4** | **Females: 11 Males: 11** |
